# Research Progress of Myocardial Fibrosis and Atrial Fibrillation

**DOI:** 10.3389/fcvm.2022.889706

**Published:** 2022-07-25

**Authors:** Guangling Li, Jing Yang, Demei Zhang, Xiaomei Wang, Jingjing Han, Xueya Guo

**Affiliations:** ^1^Department of Cardiology, Lanzhou University Second Hospital, Lanzhou University, Lanzhou, China; ^2^Department of Pathology, Gansu Provincial Hospital, Lanzhou, China

**Keywords:** myocardial fibrosis, atrial fibrillation, extracellular matrix, fibroblasts, collagen

## Abstract

With the aging population and the increasing incidence of basic illnesses such as hypertension and diabetes (DM), the incidence of atrial fibrillation (AF) has increased significantly. AF is the most common arrhythmia in clinical practice, which can cause heart failure (HF) and ischemic stroke (IS), increasing disability and mortality. Current studies point out that myocardial fibrosis (MF) is one of the most critical substrates for the occurrence and maintenance of AF. Although myocardial biopsy is the gold standard for evaluating MF, it is rarely used in clinical practice because it is an invasive procedure. In addition, serological indicators and imaging methods have also been used to evaluate MF. Nevertheless, the accuracy of serological markers in evaluating MF is controversial. This review focuses on the pathogenesis of MF, serological evaluation, imaging evaluation, and anti-fibrosis treatment to discuss the existing problems and provide new ideas for MF and AF evaluation and treatment.

## Introduction

Atrial fibrillation (AF) is the most common arrhythmia in clinical practice. Epidemiological studies have shown that 2% of people worldwide suffer from AF (1). It is estimated that by 2050, 6–12 million people will suffer from AF in the United States, and by 2060, 17.9 million people will suffer from AF in Europe (2). AF can cause heart failure (HF), ischemic stroke (IS), and other complications, increasing disability and mortality. Myocardial fibrosis (MF) is caused by an imbalance in the production and degradation of extracellular matrix (ECM), especially the excessive deposition of collagen, which in turn leads to the formation of scar tissue in the intercellular matrix. Current studies have shown that MF is involved in the pathogenesis of AF, dilated cardiomyopathy, and hypertrophic cardiomyopathy (3, 4). Therefore, it is particularly necessary to evaluate MF. Serological and imaging evaluation are the main methods to evaluate MF, but imaging evaluation of MF has some limitations. Cardiac Magnetic Resonance (CMR) may be the best method for non-invasive evaluation of MF currently, but there are many parameters for CMR evaluation and each has its limitations and is time-consuming and labor-intensive. Serological evaluation is an ideal method to evaluate MF. However, there are many serological indexes, and the accuracy of different indexes needs further study. In addition, anti-myocardial fibrosis therapy for different targets is of great significance for clinical transformation and is expected to reduce the burden of AF.

## Classification Of Myocardial Collagen And Regulation Of Myocardial Fibrosis

### Classification of Myocardial Collagen

The myocardium adapts to various injuries and stimuli through structural and functional remodeling, mainly through the excessive deposition of ECM and the hypertrophy of cardiomyocytes. Normal cardiac tissue is composed of cardiomyocytes, non-cardiomyocytes (such as endothelial cells, vascular smooth muscle cells, and fibroblasts), and ECM. The atrial and ventricular ECM accounted for 49 and 17% of the total volume of cardiac tissue, respectively (5, 6). Collagen fibers are an essential part of the ECM, and it is composed of seven collagen subtypes, among which type I collagen accounts for over 90% of the total collagen. It has higher hardness and solid tensile strength to maintain the ventricular wall tension. Type III collagen accounts for about 10% of the total collagen. It has more delicate fibers and intense elasticity and is used to maintain myocardial compliance. The decrease in diastolic heart function may be related to the decrease in the amount or proportion of type III collagen. In addition, there is a small amount of type II, type IV, type V, type VI, and type XI collagen in the cardiac ECM (7, 8). Besides, some other ECM components that are not collagen and are associated with MF [e.g., Osteopontin, Periostin, and Galectin-3 (Gal-3)]. Under pathophysiological conditions such as aging, oxidative stress, ischemia, and tissue necrosis, ECM protein is synthesized and secreted in large quantities. It increases the amount and concentration of collagen in cardiac tissue, the ratio of collagen subtypes I/III, and a disorder of collagen arrangement. These lead to the occurrence of MF, which may eventually lead to the occurrence of AF (9).

### Regulation of Myocardial Fibrosis

Cardiac fibroblasts (CF) are the main cellular component of the myocardial interstitium, accounting for about 24.3% of the atrial tissues and 15.5% of the ventricular tissues (10). It plays a vital role in maintaining the normal structure and function of the heart and is also a key regulator of pathological MF and ventricular remodeling. Normal cardiac tissue generally does not contain myofibroblasts which are derived from various cell types induced by myocardial ischemia or pressure stimulation. Myofibroblasts can repair necrotic tissue more effectively than fibroblasts (11). However, fibroblasts can transdifferentiate into myofibroblasts under the stimulation of various inflammatory mediators, cytokines, mechanical tension, and other factors. Then, they secrete more collagen fibers to promote ventricular remodeling. If they are continuously activated, myocardial interstitial fibrosis will be formed.

Under the action of monocyte chemoattractant protein, transforming growth factor beta (TGF-β), and other chemokines, bone marrow-derived fibroblasts migrate from bone marrow to cardiac tissue, transform into myofibroblasts, and secrete a large number of collagen to participate in tissue repair (12). As a critical molecule of MF, TGF-β can promote collagen synthesis, upregulate the expression of other fibrotic factors, inhibit ECM degradation, and increase ECM deposition. TGF-β can also transform fibroblasts into myofibroblasts and activate various signal transduction pathways. The Smad pathway is the principal intracellular signal transduction pathway among these pathways. The TGF-β-Smad pathway plays an essential role in the occurrence of MF (13, 14). Several studies also found that the expression of matrix metalloproteinase-2 (MMP-2), MMP-9, MMP-12, and TGF-β increase in the fibroblasts of mice chronically infected with Trypanosoma cruzi and promote the differentiation of fibroblasts into myofibroblasts (15, 16). In addition, the renin-angiotensin-aldosterone system (RAAS) is involved in the occurrence and regulation of MF, and its main pathway is the TGF-β1-Smad2/3 pathway, which promotes the increase of collagen secretion. Again, the results were confirmed in a pressure-overloaded MF model induced by angiotensin II (Ang-II) perfusion and showed that Ang-II induce bone marrow-derived CD34 + /CD45 + fibroblasts to express type I collagen (17). In addition, apelin inhibits Ang-II-induced atrial fibrosis and AF *via* TGF-β/Smad2/α-SMA pathway (18). The above evidence indicates that the TGF-β-Smad pathway is an essential pathway for myocardial collagen deposition and plays a vital role in MF.

**FIGURE 1:**
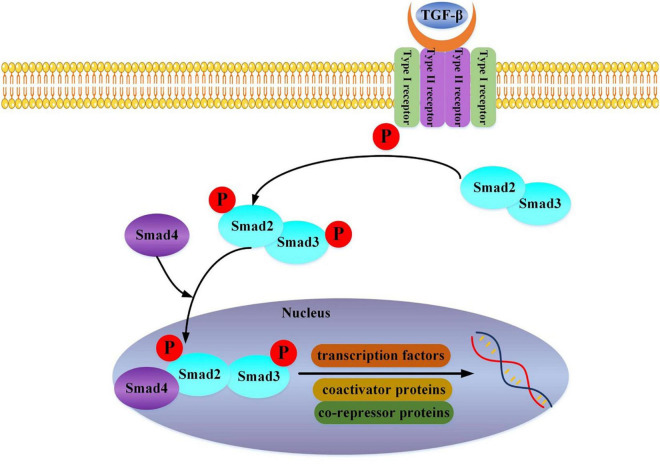
TGF-beta binds to the Type II receptor and recruits the Type I receptor, whereby the Type II receptor phosphorylates and activates Type I. The Type I receptor, in turn, phosphorylates receptor-activated Smad2 and Smad3. Then Smad2, Smad3, and Smad4 combine and translocate into the nucleus together. The interaction of the Smad complex with other DNA binding proteins (transcription factors, coactivator proteins, co-repressor proteins) activates specific gene expression.

We can find that TGF-β is a crucial link. It mainly acts in the following ways: TGF-β1 activates the classic TGF-β1-Smad2/3 pathway (Figure 1) (19), the non-classical TAK1/p38 pathway (20), and ROCK/MRTA-F pathway (21) to upregulate the expression of type I collagen gene and promote its synthesis. Further study has shown that the haploid deletion of ALK4 can reduce the susceptibility of atrial remodeling and AF in the model of pressure overload by inhibiting the activation of Smad2/3 (22). Furthermore, DLK1 is a crucial factor in differentiating fibroblasts into myofibroblasts. The loss of DLK1 leads to the down-regulation of MIR-370 and the activation of the TGF-β-Smads pathway to promote MF leading to excessive ECM deposition (23).

Further study of the expression of PAR2 and caveolin-1 through the PAR2KO mouse model and endocardial biopsy of heart failure with reduced ejection fraction (HFpEF) patients indicated that PAR2 is an essential regulatory factor of the profibrotic factor PAR1 and TGF-β pathway. The absence of PAR2 leads to the decrease of caveolin-1 expression and the increase of PAR1, promoting the formation of MF. On the contrary, antagonizing PAR1 can reduce MF by 40% (24). At the same time, a study has found that the JAK-STAT pathway might lead to left atrial fibrosis, which indicates that JAK-STAT pathway inhibitors, such as S3I-201, may become a new therapeutic approach for AF. Moreover, more studies are needed to verify the long-term effect of S3I-201 (25). Upregulation of interleukin 11 (IL-11) is a primary transcriptional response to TGFβ1 exposure. IL-11 and its receptor (IL11RA) are specifically expressed in fibroblasts, where they drive atypical ERK-dependent autocrine signaling, which is required for fibrin synthesis (26).

However, we need to point out that the relationship between fibrosis and fibrosis-generated factors is quite complicated, particularly in HF. There is upregulated expression of fibrosis-associated markers in atrial tissues isolated from mild or median but not severe systolic HF. A similar trend has been reported in the case of TGF-β1, which is robustly expressed after 1–7 days of pacing in dog, but less so after that in atria (27, 28).

### The Role of Profibrogenic Factors in Inflammation/Oxidative Stress

Profibrogenic factors and inflammatory/oxidative stress are also involved in MF. Fibrosis-promoting factors include Ang-II, aldosterone, catecholamines, connective tissue growth factor (CTGF), endothelin (ET), platelet-derived growth factor (PDGF), and reactive oxygen species. Their primary mechanism is also through the promotion of fibroblasts transforming into myofibroblasts. Myofibroblasts produce twice as much ECM as fibroblasts and secrete some biologically active substances to promote the development of fibrosis, forming positive feedback loop, which accelerate the development of MF (8). A study found that overexpression of myocardial-specific PDGF in transgenic rats can also lead to MF (29). Several other studies found that the profibrotic CD163+ M2 macrophages in atrial tissue are related to the expression of procollagen. The CD163+ M2 macrophages in AF patients are more than in sinus rhythm patients, suggesting that inflammation induced by CD163+ M2 macrophages may be involved in the occurrence of MF and AF (30). They release chymotrypsin and tryptase by degranulation to increase the expression of TGF-β and promote MF (31–33). Inflammatory cell infiltration in atrial tissue of patients with lone AF confirmed that inflammatory reaction could promote oxidative damage to atrial tissue and persistence of AF (34). When IL-10 is significantly reduced in patients with AF, IL-6 is significantly increased. IL-6 can inhibit regulatory T cells’ function and increase the expression of α-SMA, type I collagen, and type III collagen, further leading to MF (35). In addition, the increase in myocardial inflammation in transgenic mice leads to interstitial collagen accumulation (36). Moreover, oxidative stress leads to substrate for triggered activity and reentry in the posterior wall of the left atrium in patients with HF through CaMKII, which is independent of MF and complementary to MF (37). Studies have shown that C-reactive protein induce cardiac injury through inflammatory reaction and serum complement activation (38). Reactive oxygen species can also induce cardiac fibroblasts (CF) to differentiate into myofibroblasts and promote the occurrence of MF (39). The above evidence indicates that inflammation and oxidative stress are involved in the formation of MF.

### The Role of Non-coding RNA

Most genes in the human genome can be transcribed, but only 1–2% can translate protein, called coding RNA. Most transcribed sequences do not encode proteins and are called non-coding RNA (ncRNA). It can perform its biological functions at the RNA level, mainly including small nucleolar RNA (snoRNA), small nuclear RNA (snRNA), microRNA (miRNA), circular RNA (circRNA), long non-coding RNA (lncRNA), and small interfering RNA (siRNA). They play an essential role in regulating atrial structure remodeling and MF in patients with AF. The overexpression of miR-27b-3p can regulate the Wnt/β-Catenin signaling pathway reducing the occurrence and duration of AF, reducing MF and increasing the expression of connexin 43, and also reducing the expression of collagen-I, α-SMA, Collagen-III, TGF-β1, Wnt3a, and p-β-catenin (40). Besides, lncRNA plasmacytoma variant translocation 1 (PVT1) can be expressed through the miR-128-3p-sp1-TGF-β1-Smad pathway to promote MF in mice and humans (41). At the same time, the clustered miR-23b-3p and miR-27b-3p can promote MF by acting on the TGFBR3-Smad3 signaling pathway (42). On the other hand, overexpression of microRNA-30c (miR-30c) in CF can inhibit cardiac fibroblasts’ proliferation, differentiation, migration, and collagen production. And transfection of adeno-associated virus 9 (AAV9)-miR-30c into the inferior vena cava of mice can alleviate left atrial MF. Further, the opposite result was observed by reducing the expression of miR-30c (43). Besides, miR-210 can inhibit the function of regulatory T cells and promote MF by targeting Foxp3 (35). Some research suggests that overexpression of miR-21 in CF can upregulate the phosphorylation of STAT3, increase the expression of MF-related genes and then promote the occurrence of AF (44). Simultaneously, miR-21 is also related to the outcome of patients with persistent AF after catheter ablation (45).

In addition, circRNA-000203 is significantly increased in CF induced with Ang-II. Overexpression of circRNA-000203 primarily increase the expression of collagen 1A2, collagen 3A1, and α-SMA in CF. Furthermore, dual fluorescent gene assays showed that circRNA-000203 inhibit the anti-fibrotic effect of miR-26b-5p by attenuating the interaction of miR-26b-5p with CTGF and collagen 1A2 (46). Related circRNAs also include circRNA-010567, circNCX1, circHIPK3, circNFIB, and circFndc3b, all of which are involved in the regulation of MF. Meanwhile, studies have shown that lncRNA CHRF is significantly elevated in the myocardial tissue of transverse aortic constriction (TAC) model mice.

Further studies found that CHRF can act as an endogenous “sponge body” to adsorb miR-489. Due to the inhibition of adsorption, the expression of miR-489 is reduced, then regulating cardiac hypertrophy. Conversely, in the myocardial tissue of mice with higher expression of miR-489, it was found that the degree of fibrosis is more significantly reduced (47).

The above evidence all represents the correlation between non-coding RNA and MF. It provides help for our understanding of the pathogenesis and regulation mechanism of MF. The most important thing is that these potential regulatory pathways have clinically transformative significance. The relationship between non-coding RNA and MF is shown in Table 1 (40, 41, 43, 44, 46, 48–62).

**TABLE 1:** Studies of the relationship between non-coding RNA and myocardial fibrosis.

Types of non-coding RNA	Target of action	Role of myocardial fibrosis
miRNA miR-10a (49) miR-17 (50) miR-21 (44, 51) miR-27-b (52) miR-27-b-3p (40) miR-23b-3p and miR-27b-3p (42) miR-29b (53) miR-30c (43) miR-133 (54, 55) miR-210 (35) miR-146b-5p (56) lncRNA lncRNA-NRON (57, 58) lncRNA-PVT1 (41) lncRNA-PCAT1 (48) lncRNA-MIAT (59) circRNA circRNA-NFIB (60) circRNA-000203 (46) circRNA-010567 (61, 62)	TGF-β 1/Smads TGF-β 1/Smad7 STAT3 ALK5/Smad2/3 Wnt3a and Wnt/β -Catenin TGFBR3-Smad3 COL1A1 and COL3A1 TGF-β RII CTGF-mRNA Targeting Foxp3 TIMP-4 NFATc3 TGF-β 1/Smads TGF-β 1 miR-24/TGF-β 1 miR-433 miR-26b-5p/Col1a2, CTGF miR-141/TGF-β 1	+ + + - - + - - - + + - + + - - + +

*“+” indicates increased myocardial fibrosis; and “-” indicates reduced myocardial fibrosis.*

## Myocardial Fibrosis Leads To Atrial Fibrillation

### Myocardial Fibrosis Is the Matrix of Atrial Fibrillation

MF is a hallmark of structural remodeling of AF. Studies showed that collagen content in the atrial muscle of patients with isolated AF is significantly increased compared with the control group of sinus rhythm. The increase in collagen can enhance heterogeneity of myocardial conduction and cardiac electrical instability and make it easier to form reentry, which increases the susceptibility to AF (39). Moreover, the more fibrous tissue in the myocardium, the more accessible AF is sustained, suggesting that MF can provide the matrix for the occurrence and maintenance of AF. The AF susceptibility increase in the rat model of MF induced by isoproterenol, which indicates that MF is one of the primary substrates of AF (63). At the same time, studies have shown that uremic toxins can cause MF through oxidative stress, leading to AF (64, 65). Cardiomyocyte-restricted overexpression of FKBP12 decreased atrial Nav1.5 expression levels and mean peak INa in transgenic (αMyHC-FKBP12) mice, which is associated with increased peak L-type Ca^2+^ currents and MF. Electrophysiological and structural changes promote the development of focal conduction block and alter action potential duration and spontaneous AF (66). In clinical congestive HF models induced by rapid atrial and ventricular pacing, MF has similar pathophysiological characteristics. For example, fibrosis can cause a delay of local atrial conduction and increase conduction heterogeneity, leading to reentrant and focal atrial arrhythmias (67, 68). Besides, whole exon sequencing was performed in 24 families with at least three members diagnosed with AF. It was found that the expression of titin truncating variants (TTNtv) is increased in patients with AF. In addition, the zebrafish model modified by CRISPR/cas9 with TTNtv shows a higher level of MF. These pieces of evidence support the theory that MF promotes the occurrence of AF (69).

In the rat model after myocardial infarction (MI), it was found that the duration of AF is significantly correlated with the level of MF (70). There was increased interstitial fibrosis in elderly mice compared with young mice, and the regulatory factors of ECM remodeling are also changed (71). The study also found that interstitial fibrosis is different in the different health statuses of the same-age mice. Similarly, mitochondrial dysfunction can lead to the aggravation of age-dependent MF and promote the occurrence of AF (72). Besides, long-term rapid atrial pacing can lead to the accumulation of ECM protein in atrial muscle. It also observed that AF can lead to MF (73, 74).

Further research found that AF can promote the transformation of mesenchymal stem cells in the atrium to pro-fibrosis phenotype (75). The above evidence shows that MF and AF may influence each other, and form a vicious circle. Then, the disorder of collagen arrangement can separate the cardiomyocytes, making the electrical conduction between the cardiomyocytes abnormal, which provides a substrate for the occurrence of AF (9). However, there are some different perspectives. AF occurrence is usually associated with increased MF, but AF, including persistent AF, can readily occur without increased fibrosis (76, 77). Severe MF is associated with severe depression of atrial excitability, reducing AF occurrence (78). Note that atrial structural remodeling and fibrosis are significantly greater in HFrEF vs. heart failure with preserved ejection fraction (HFpEF) patients, but AF prevalence is more significant in HFpEF patients. Moreover, HF patients with ischemic heart disease (IHD) etiology commonly have a greater atrial structural remodeling vs. HF patients with hyperparathyroidism (HPT) etiology, but AF prevalence is much lower in HF patients with IHD etiology (79, 80). The explanation for these results needs to be further explored in the future.

### Mechanism of Atrial Fibrillation

When the preload of the heart increases or inflammation occurs, fibrin is deposited between the cardiomyocytes, the cardiomyocytes are not lost, and fibrotic scars are formed, which is called reactive fibrosis. In this case, the cardiomyocytes are separated by fibrous tissues, causing electrical conduction obstacles, increasing the anisotropy of electrical conduction, forming “zigzag” conduction, and then forming small reentrants (4, 81). When cardiomyocytes undergo apoptosis or necrosis, fibrous tissue proliferates to replace these tissues, a process called reparative fibrosis. The formation of fibrous tissue will cause a unidirectional block of electrical conduction and form a large reentry (67). Besides, in patients with persistent AF, the intensity of late gadolinium-enhanced magnetic resonance imaging (LGE-MRI) is related to myocardial conduction velocity, indicating that MF can lead to myocardial conduction heterogeneity (82). Therefore, MF can cause AF by interfering with the continuity of myocardial bundle conduction. However, there is another solid opposing argument for a “fibrosis-AF” causative connection: The whole story of fibrosis involvement in AF is related to reentry. It used to be “established” that reentry is the prime mechanism of AF. Nowadays, with the improvement of mapping technologies, more researchers do not record reentry at all (83–85). Moreover, those who consistently record reentry employ the phase mapping techniques for reentry detection, which, as recently reported, has a very low specificity for reentry identification (86). These results challenge several established views about the occurrence of AF and also point the way to future research.

The heart is a syncytial body. There are gap junctions between the myocardial cell, which increases the stability of the electrical conduction of the myocardial cells, reduces the impedance between the myocardial cells during the conduction process, and makes the myocardial cells exhibit all or nothing contraction characteristics. Connexin 40 (Cx40), connexin 43 (Cx43), and connexin 45 (Cx45) are the significant components of gap junctions in the myocardium, and Cx43 is the major component of gap junctions in the ventricular myocardium. The study indicates that the loss of Cx43 can lead to arrhythmia. It is pointed out that JNK can inhibit its transcriptional activity and down-regulate the expression of Cx43 mRNA by enhancing the binding between c-jun and Cx43 promoter, which leads to abnormal conduction of myocardium (87). However, silencing EDH1 leads to the damage of Cx43 internalization, thus protecting the coupling of gap junction communication between cardiomyocytes and the stability of conduction (88).

Additionally, the expression of Cx40 in a goat AF model is substantially reduced, and the recovery of Cx40 is comparatively slow after AF converted to sinus rhythm demonstrating that Cx40 is related to AF (89, 90). Further research found that the deletion of ALK4 haploid alleviate the decrease of Cx40 and the redistribution of Cx43 from the intercalated disc to the lateral membrane, thus improving the local conduction abnormality and inhibiting the occurrence of AF (22). In addition, in C57BL/6 mice with metabolic syndrome, very low density lipoprotein (VLDL) can slow down the conduction between the myocardium and leads to AF by reducing the expression of Cx40 and Cx43 and glycosylating the serine of Cx40 and Cx43 (91, 92).

There is evidence that both fibroblasts and myofibroblasts interact with cardiomyocytes in electrical coupling forms, which is particularly common in myofibroblasts (93, 94). Vitro experiments have shown that myofibroblasts in fibrotic remodeled myocardium and infarcted myocardium scar tissue can electrically interact with cardiomyocytes through gap junctions (95, 96). Furthermore, mathematical modeling to study fibroblast-cardiomyocyte interactions reveal two critical determinants of the resulting electrical interactions: the number of coupled fibroblasts per cardiomyocyte and the closeness of the electrical coupling between cardiomyocytes and fibroblasts (97). Both fibroblasts and myofibroblasts are small cells with sparse cytoplasm. Histological studies alone are challenging to identify Cx43 and tunneling nanotubes (TNTs), which are important structures regulating the function between cardiomyocytes and fibroblasts/myofibroblasts (98). Abnormal spatial distribution, structure, and quantity of Cx43 can affect the function of electrical coupling and metabolic coupling of gap junctions, leading to arrhythmia. Some researchers used a genetically encoded voltage-sensitive fluorescent protein 2.3 (VSFP2.3) to monitor the transmembrane potential of cardiomyocytes and non-cardiomyocytes and recorded cardiomyocyte-like action potentials in non-cardiomyocytes in the healed border zone of cryoinjury, which provides strong evidence for the existence of heterogeneous intercellular electrical coupling (99). The study also inferred that TNTs might be the underlying structural basis for electrical coupling. However, some studies have also shown that there is no formation of TNTs between CF and cardiomyocytes, and there is no mitochondrial transfer (100). Therefore, the specific situation needs further research.

Recent studies have shown that the interaction between cardiomyocytes and fibroblasts plays an essential role in AF. In addition to producing ECM, fibroblasts can also secrete various substances to mediate the interaction between fibroblasts and cardiomyocytes. For example, fibroblasts contain primary cilia to interact with PC1 protein and participate in TGF-β1-smad3 activation and ECM production (101). The miR-370 in cardiomyocytes can inhibit the TGF-β pathway (23). In terms of miR-370, its mode of action is still unclear. Cardiomyocytes may secrete miR-370 in a paracrine manner through exosomes, which act on fibroblasts.

Moreover, the cocultured neonatal mouse fibroblasts can prolong the action potential duration and slow down the conduction velocity of myocardial cells by producing paracrine factors acting on the ion channels on the myocardial cell membrane, and all of these changes jointly lead to the occurrence of the rotor reentry in the atrium (93, 102, 103). On the other hand, when the pressure load increases, the cardiac fibroblasts are pulled, which can directly cause the electrical instability of cardiac myocytes and then promote the occurrence of AF, which is called mechanical-electrical feedback (104). In the same case, the stretch of cardiomyocytes can produce Ang-II, leading to the activation of fibroblasts and promoting collagen secretion. Other research has reported that TGF-β1 expression in palmitate-induced insulin-resistant neonatal cardiomyocytes and atrial fibroblasts *in vitro* is significantly higher than in the control group (105).

## Evaluation Of Myocardial Fibrosis

### Serological Markers

#### Transforming Growth Factor Beta

TGF-β is a cytokine that undertakes multiple functions and participates in cell proliferation, apoptosis, and migration. Its overexpression can lead to MF. Robust evidence showed that the expression of the MF marker TGF-β is increased after infusion of Ang-II in WT and ATF3-KO mice (106). The study observed increased expression of Tgf-βR2 and elevated serum levels of TGF-β1 in Jak^2VF/ + −^ Hmga2 mice that exhibited MF (107). The selective knockout of TGF-β receptors Tgfbr1/2, Smad2, or Smad3 in the mouse line showed that Tgfbr1/2, Smad2, or Smad3 could promote the programmed expression of fibrosis genes and the remodeling of the ECM (105, 108). At the same time, Ang-II can induce TGF-β expression and increase ECM (109). Further research found that MF can be alleviated by inhibiting TGF-β/Smad3 and GSK-3β pathways (110). The above evidence shows that TGF-β is associated with MF and has the potential to be a serological marker of MF. We can monitor MF by measuring the content of TGF-β in the serum.

#### Matrix Metalloproteinase

MMPs are involved in the metabolism of ECM. MMPs are a large family, so named because they require metal ions such as Ca^2+^ and Zn^2+^ as cofactors. MMPs can degrade various protein components in the ECM, destroy the histological barrier, and play a vital role in the formation of MF, so they have received increasing attention in the formation of MF. Studies have shown that MMP-2 knockout mice may reduce MF by decreasing macrophage infiltration after MI (111). In rats after MI, the increase of tissue inhibitor of MMP-1 and the decrease of MMP-9 can ameliorate left ventricular MF (112). However, studies have shown that MMP-mediated ECM remodeling is necessary for organ formation in the Drosophila model. It can also prevent excessive or ectopic ECM protein assembly during growth (113). The study is different from the above results, which may be explained by different models, or the regulation of MMP is a two-way process that plays different roles in different stages of tissue and organ development. In addition, endogenous osteopontin can lead to inflammatory response, cardiac hypertrophy, cardiac remodeling, and interstitial fibrosis by upregulating the expression and activity of CCL5/MMP-2 (114). Simultaneously, membrane type 1-matrix metalloproteinase (MT1-MMP) plays a crucial role in MF and matrix remodeling in the mouse model of pressure overload (115). On the other hand, the reduction of MMP-9 regulates the amount of myocardial collagen by inhibiting the TGF-β pathway, reducing the expression of periosteal protein and CTGF (116). Further research found that in the mouse model after MI, macrophage-derived MMP-9 can increase the aggregation of collagen but reduce the cross-linking between the collagen (117). Tissue inhibitors of metalloproteinases (TIMPs) can cooperate with MMPs to regulate ECM production. TIMP-1 is an inhibitor of MMP-9; it forms an enzyme-inhibitor complex with MMP-9, which prevents MMP-9 from degrading ECM (118). Some studies also showed that MMP-1, MMP-2, and MMP-14 decrease with the increase of age, while TIMP-1 increase (119). However, studies have shown no significant difference in TIMP-1 in persistent AF patients and sinus rhythm patients (9). From the above studies, the role of TIMP-1 in the regulation of myocardial collagen is controversial. The possible explanation is that TIMP-1 increases when stimulated by acute stress, such as changes in left atrial volume. It tends to be stable after entering the chronic remodeling phase. The expression of MMP-28 in mice will be upregulated with the aging of the myocardium, and in MMP-28^–/–^ mice after MI, the levels of inflammatory response and ECM decrease, indicating that the increase of MMP-28 will lead to more ECM production (120, 121). In human transplanted heart, the transcription levels of MMP-14, TIMP-1, MMP-2, and MMP-9 strongly correlate with the expression of myocardial collagen (122, 123). In addition, in the diabetic rat model, reducing the expression of MMP-2 and MMP-9 can reduce MF (124). The above evidence indicates that MMPs are involved in the formation of MF. Among the known MMPs, most of them promote the formation of MF. The common ones are MMP-14, MMP-2, MMP-8, and MMP-9, and detection of their expression levels may reflect MF to some extent.

#### Galectin-3

**FIGURE 2:**
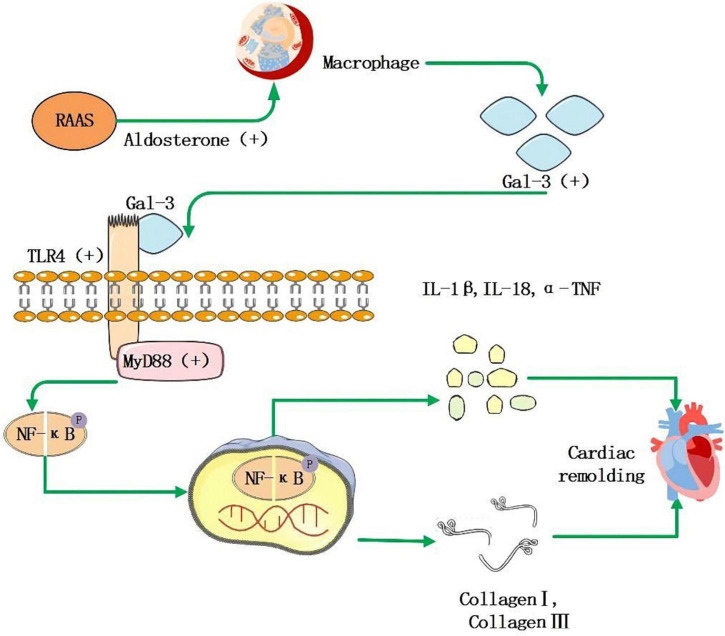
RAAS system activation leads to increased secretion of aldosterone, which stimulates macrophages to secrete increased amounts of Gal-3, and it acts as a ligand to bind to TLR-4, further activating downstream MyD88, NF-κB signaling pathway that facilitates the increase of inflammatory factor IL-1β, IL-18, α-TNF, and collagen I, collagen III, then leads to myocardial remodeling and fibrosis.

Galectin, a 29–35 kDa protein, belongs to the lectin family, and it is involved in many biological processes as a galactoside binding protein. Many organs, including the heart, express Gal-3. Its essential role in MF is to promote the transformation of quiescent fibroblasts into myofibroblasts and produce and secrete matrix proteins, including fibrin and fibronectin. Gal-3 is not only involved in the production of collagen but also in the maturation and cross-linking process of collagen. Finally, it combines with matrix protein to achieve MF; Gal-3 can be combined with other Gal-3 residues to form a dimer and a network structure, which makes ECM accumulate, causes the reconstruction of cardiac structure and promotes the development of AF. As shown in Figure 2, the expression of Gal-3 is related to the increase of macrophages, which increases the activity of fibroblasts, and leads to ECM accumulation (125, 126). In the study of Hernandez-Romero et al. (127), high Gal-3 is an independent predictor of atrial appendage fibrosis. Gal-3 upregulation induced by Ang-II and aldosterone has also been implicated in fibrotic responses following activation of the RAAS, emphasizing its central role in MF (126, 128). Besides, Gal-3 may be involved in atrial structural remodeling, further contributing to progressive fibrosis in AF patients (129). However, in the CRIC study (130), there was no significant correlation between Gal-3 levels and AF events in chronic renal disease patients. Besides, several findings suggest that despite excess cardiac and systemic Gal-3 in HF patients of hypertensive origin, this molecule is not associated with histological, molecular, and biochemical parameters associated with MF (131). These clinical studies are limited by small sample sizes and poorly detailed assessments. A meta-analysis of the association between baseline circulating Gal-3 levels and recurrence of AF in catheter ablation patients found that Gal-3 levels at baseline are more significant in patients with recurrent AF than in patients without recurrent AF (132). In conclusion, Gal-3 remains a potential serological marker for predicting MF. More critically, serum levels of Gal-3 can be reliably determined by a commercially available enzyme-linked immunosorbent assay (ELISA).

#### Endothelin-1

Endothelin-1 (ET-1) is a vasoactive peptide, and its primary function is to constrict blood vessels. When left atrial pressure increases or atrial myocyte is hypertrophic, atrial myocyte, fibroblasts, and vascular smooth muscle cells can secrete ET-1 to promote left atrial remodeling and produce direct toxicity for cardiomyocytes, resulting in cardiomyocyte hypertrophy and ECM production. ET-1 is essential in MF, and ET-1 expression is markedly upregulated in mouse CF (133, 134). Studies also found that transcardiac gradients of plasma ET-1 correlated with plasma levels of type III amino-terminal peptide procollagen, a marker of MF (135). Besides, a high plasma ET-1 is an essential predictor of AF recurrence after surgical treatment (136).

#### Collagen Peptides

Fibroblasts take up the required amino acids, such as proline (Pro) and lysine (Lys), synthesize the proalpha polypeptide chain on the nucleoprotein body of the rough endoplasmic reticulum, and transport the polypeptide chain after reaching the Golgi complex; then procollagen is formed. There are three main types of procollagens involved in MF: type I procollagen carboxy-terminal propeptide (PICP), type III procollagen amino-terminal propeptide (PIIINP), and procollagen type I amino-terminal propeptide (PINP). Moreover, I collagen C terminal telopeptide (ICTP) is also involved in MF. ICTP reflects the degradation rate of type I collagen, PIIINP reflects the transformation of type III collagen, and ECM is mainly composed of type I and type III collagen. Under physiological conditions, only a very small amount of procollagen peptides maintain the structure of ECM, and most of the procollagen peptides are degraded. Under pathological conditions, the degradation of type I collagen decreases, resulting in the imbalance of I and III collagen proportion, leading to MF. The serum levels of ICTP and PIIINP in the AF recurrence group and AF group were higher than those in the sinus rhythm Group (137), suggesting that they can predict the occurrence and the recurrence of AF. Similarly, ICTP and PIIINP can be used as circulating markers of MF, predicting the recurrence of AF (138). Further research found type I collagen cross-linking (CCL+) and type I deposition (CD+) patients had a higher incidence rate of AF and the recurrence rate after catheter ablation (139). In addition, baseline PIIINP and ICTP are positively related to incident AF (140). The above evidence suggests that collagen peptides, especially PIIINP and ICTP, can serve as MF and AF serological markers.

### Imaging Evaluation

#### Evaluation of Echocardiography

Speckle tracking echocardiography (STE) was used to evaluate ischemic MF. Sakurai et al. (141) studied the echocardiographic manifestations of acute coronary occlusion and reperfusion in dogs and analyzed the circumferential strain and radial strain; the peak systolic and end-systolic circumferential and radial strains in the risk area of coronary artery occlusion decrease significantly, and the ventricular wall return to the baseline level after reperfusion. Several studies have found that applying end-systolic radial strain peak to reflect the formation of the segmental myocardial scar has very high sensitivity and specificity (142, 143). Therefore, the comprehensive application of 2D-STE strain parameters can better describe the characteristics of myocardial dysfunction in different stages of myocardial ischemia and can define segmental scars.

Tissue Doppler imaging (TDI) and pulse wave Doppler myocardial imaging evaluated the correlation between isovolumic systolic velocity loss and transmural scar after MI. The results show that the loss of isovolumic contraction velocity in the abnormal segment of the ventricular wall has high sensitivity but low specificity. Therefore, TDI to evaluate isovolumic systolic velocity may not be suitable for the clinical detection of transmural scar (144).

Myocardial elastography (ME) technology based on strain imaging can map the full-thickness strain tensor distribution of the ventricular wall, has no angle dependence on the standard two-dimensional ultrasound slice, and has a high temporal and spatial resolution. Lee et al. (145) completed the dynamic observation of left ventricular full-thickness strain imaging when the coronary blood flow of the canine heart decrease from 0 to 100%. This study shows that the danger zone gradually expands with the gradual decrease of coronary blood flow. Although it is theoretically speculated that MF can significantly reduce myocardial elasticity, there is little reports on the evaluation of MF by ME.

In evaluating non-ischemic MF, 2D-STE: Kramer et al. (146) used two-dimensional STE to evaluate the regional myocardial deformation of MF in Fabry disease and found the presence of cardiac magnetic resonance late gadolinium enhancement (CMR-LGE). Patients with CMR-LGE show lower overall systolic longitudinal strain than those without CMR-LGE, and patients with severe CMR-LGE show lower overall systolic longitudinal strain than those with mild or no CMR-LGE. Hoffmann et al. (147) compared 2D-STE with CMR-LGE to evaluate MF in patients with severe aortic stenosis. The peak systolic longitudinal strain of 2D-STE increase from basal segment to apical segment, but there is no difference in the peak systolic circumferential strain. There is a high degree of negative correlation between the peak longitudinal strain of the left ventricle during systole and the amount of MF measured by CMR-LGE. Therefore, STE can accurately identify the degree and the location of fibrosis in non-ischemic MF. However, its measurement value depends on the load, which may limit its application in some aspects.

#### Evaluation of Cardiac Magnetic Resonance Imaging

In the assessment of ventricular fibrosis, due to the high resolution of magnetic resonance imaging (MRI), delayed enhancement of MRI is more advantageous than other methods in assessing myocardial tissue activity, and it is the gold standard for non-invasive assessment of myocardial tissue activity (148). Most research on the correlation between MF and cardiovascular disease uses MRI to detect MF. However, the traditional MRI delayed enhanced imaging requires normal myocardium as the control after injection of the contrast agent to display the fiber tissue of the remaining contrast agent. Therefore, the traditional MRI can only detect focal MF. Studies have shown that in patients undergoing heart transplantation or surgical removal of part of the myocardium from hypertrophic cardiomyopathy, compared with a histopathological examination, the area of MF detected by delayed CMR enhancement is significantly reduced, indicating that MRI may underestimate the degree of MF. T1 mapping is a quantitative measurement of diffuse MF. The main parameters are T1 value before enhancement, T1 value after enhancement, and extracellular volume (ECV) (149). The T1 value before myocardial enhancement reflects the comprehensive signal of the volume of myocardial cells and ECM, and the ECV reflects the ratio of the ECM to the volume of the left ventricle. At present, it is considered that the T1 value after enhancement is affected by many factors, such as heart rates and glomerular filtration rate. Its accuracy and repeatability are not as good as the pre-enhancement T1 value. Therefore, the pre-enhancement T1 value and ECV are used as the evaluation indexes of MF. Compared with pathological results, the relevant parameters of T1 mapping can accurately evaluate the degree of MF. The study by Ambale-Venkatesh et al. (150) has shown that lower T1 value and higher ECV significantly predict MF and are associated with cardiovascular events such as AF. The study by Zhao et al. (151) proved that the quantitative measurement of MF by T1 mapping can effectively predict cardiovascular adverse events in patients with HF and AF. At the same time, studies have shown that the myocardium in patients with AF presents diffuse fibrosis (152–154), and T1 mapping can better evaluate diffuse fibrosis of the myocardium. Therefore, it can be used to detect MF, which can help understand the pathophysiology of AF and predict the occurrence of adverse events in patients with MF and AF.

In the evaluation of MF, the increase in ECV and capillary reduction caused by MF cause the elution delay and the concentration increase of the contrast agent, and the contrast agent can shorten the T1 relaxation time. Hence, the fibrotic tissue has a higher signal than normal tissue. Gadolinium is commonly used as a contrast agent in delayed-enhanced magnetic resonance imaging (DE-MRI), also known as LGE-MRI. Mewton et al. (155) confirmed the correlation between the left atrial enhancement area identified by LGE-MRI and the fibrosis tissue in the left atrial surgical biopsy specimens. Spragg et al. (156) included ten patients who underwent secondary ablation after AF recurrence and found that the enhancement area of MRI has a good correlation with the low voltage area of the left atrium. In the study of Quail et al. (157), the myocardial volume of the left atrium with LA LGE ≥ 10% is significantly correlated with new atrial arrhythmia. However, different from the above results, in the cross-study, there is no significant correlation between the post-ablation atrial scar (PAAS) index measured by DE-MRI and the recurrence of AF (158). Corview was used for image processing and analysis in the DECAFF study (159). The software can realize the separation of the left atrial wall, the judgment of fibrosis, and the output of the three-dimensional model. According to the volume ratio of fibrosis area and left atrium, LGE-MRI can be divided into four stages: Utah stage I < 10%, Utah stage II 10 ∼20%, Utah stage III 20∼30%, Utah stage IV ≥ 30%, It also provides the possibility of the individualized treatment strategy for patients with AF. Moreover the more serious the Utah stage, the higher the incidence of cardiovascular and cerebrovascular events, especially strokes and transient ischemic attacks, indicating that MF is related to the increased incidence of cerebrovascular and cardiovascular events (160).

In addition, in recent studies, epicardial adipose tissue (EAT) may also indirectly evaluate MF. In chronic inflammatory diseases, the epicardium becomes the site of lipogenesis disorders, secreting pro-inflammatory adipocytokines, leading to atrial and ventricular fibrosis. The release of adiponectin is reduced and replaced by the synthesis of pro-inflammatory adipokines in the fat depot, which promotes the infiltration of macrophages, destroys the microvascular system, and activates the fibrotic pathway (161, 162). EAT can be detected by CMR imaging, but the relationship between EAT thickness and MF remains unclear. Combined with T1mapping and EAT, it may have more advantages in evaluating the prognosis of MF.

## Anti-Myocardial Fibrosis Therapy

MF plays a key role in the occurrence and maintenance of AF, so anti-fibrosis therapy has gradually become a hot issue in AF treatment. Because it is difficult to reverse the fibrosis once it is formed, it is necessary to intervene in the early stage of MF. It was found that early intervention of drugs such as pirfenidone, pioglitazone, and resveratrol can reduce MF and effectively reduce the incidence of AF (163–165). Some studies (166, 167) also confirmed that the TGF-β gene expression inhibitor, pirfenidone, can significantly reduce the AF-induced rate in congestive HF dogs by inhibiting MF. Besides studies have shown that metoprolol can alleviate MF caused by chronic obstructive sleep apnea (OSA), and its mechanism is to inhibit fat factor production by EAT (168), which may provide the basis for the treatment of MF and related cardiovascular diseases in OSA patients. In addition, ANO1 can inhibit the TGF-β/Smad3 pathway to inhibit MF in mice with MI (169). RRFR tetrapeptide activates TGF-β pathway in the TAC mouse model, increasing the expression of the samd2/samd3 pathway to accelerate MF, but it can be blocked by the TGF-β-neutralizing antibody (NAB), which provides a new target for the treatment of MF (170). In traditional Chinese medicine, Si-Miao-Yong-An decoction can inhibit TGF-β and interfere with MMP expression to inhibit MF (171). Salvianolic inhibit MF by controlling collagen deposition mediated by inhibiting the TGF-β1-Smad2/3, TXNIP/NLRP3 pathway, inflammatory IL-1β, and IL-18 (172). In diabetic rats, berberine can reduce MF by lowering IGF-R expression (124).

Moreover, the plasminogen activator inhibitor type I (PAI-1) is an inhibitory factor of MF. It has the potential to be a target for the treatment of MF, even if its early transcription promotes MF (109). The inhibitor of tissue non-specific alkaline phosphatase (TNAP) is through AMPKTGF-β1/smads and the p53 signal pathway to inhibit MF (173). Simultaneously, sodium valproate can improve myocardial remodeling and MF by reducing the expression of histone deacetylases and can delay the occurrence of AF in the rat model (174). Inhibiting the overexpression of the voltage-dependent anion channel 1 (VDAC1) can reduce atrial fibrosis, and its inhibitor VBIT-4 can decrease aldosterone-induced MF, but its role in other pathological background needs further study (175). In addition, the chronic stimulation of the sigma-1 receptor can reduce the susceptibility of atrial remodeling and AF and may become a potential therapeutic target for MF and AF (176). LTBP2 can be used as a marker of MF and a potential therapeutic target (177). Studies have shown that IL-11 is involved in an atypical pathway to promote fibrosis, which is in the priority position in the occurrence of fibrosis and can be used as a new treatment target (26). Mitoquinone can inhibit TGF-β interaction with mitochondria in mice, reducing MF caused by stress overload (178). Exchange protein activated by EPAC can improve cardiac function and reduce atrial fibrosis after MI. SP-8-pCPT, an agonist of EPAC, can reduce fibrosis after MI (179). In diabetic rabbit models, allopurinol can treat MF and AF caused by diabetes by inhibiting xanthine oxidase (180). In addition, statins and other drugs can inhibit MF through anti-inflammatory and antioxidant effects. However, the clinical indications of the drugs mentioned above need to be further clarified. In addition to anti-fibrosis treatment, the following studies also provide new ideas for anti-fibrosis treatment. Fan et al. (181) found that the developed MMP-2 inhibitor delivery system can specifically transport drug inhibitors to the MI location to improve the myocardial remodeling mediated by MMP-2. Renal denervation can also significantly reverse the electrical and structural remodeling of the atrium and inhibit atrial fibrosis (182). The above evidence shows that drugs acting on related targets or drug delivery systems and denervation surgical treatments have a particular inhibitory effect on MF. This also suggests that finding feasible targets for drugs research and development or changing the mindset to transfer drugs to target organs may be the future treatment direction for MF.

## Conclusion

MF plays a vital role in the occurrence and maintenance of AF. Many mechanisms cause MF, but the process of transforming fibroblasts into myofibroblasts is fundamental. In addition, cardiac ultrasound and CMR can assess MF, but CMR may be a better choice; the combination of T1mapping and EAT in CMR is expected to improve the accuracy of MF assessment, and relevant clinical studies can be conducted to confirm its value. Serological evaluation has great potential because of its convenience and economy, but further research is needed to find a suitable marker. In addition, the drugs development for inhibitory targets of MF may be the future research direction of MF treatment. Those changes are expected to reduce the burden of AF.

## Author Contributions

GL summarized the figure. XG corrected the manuscript. JY checked all the references. All authors wrote the manuscript and approved the final version of the manuscript.

## Conflict of Interest

The authors declare that the research was conducted in the absence of any commercial or financial relationships that could be construed as a potential conflict of interest.

## Publisher’s Note

All claims expressed in this article are solely those of the authors and do not necessarily represent those of their affiliated organizations, or those of the publisher, the editors and the reviewers. Any product that may be evaluated in this article, or claim that may be made by its manufacturer, is not guaranteed or endorsed by the publisher.
